# Arthropods dataset from different genetically modified maize events and associated controls

**DOI:** 10.1038/sdata.2018.19

**Published:** 2018-02-20

**Authors:** Zoltán Pálinkás, Mihály Zalai, Ágnes Szénási, Zita Dorner, József Kiss, Samuel North, Guy Woodward, Adalbert Balog

**Affiliations:** 1 Institute of Plant Protection, Faculty of Agriculture and Environmental Sciences, Szent István University, Gödöllő, Hungary; 2 Department of Life Sciences, Faculty of Natural Sciences, Imperial College London, Silwood Park, Berkshire SL5 7PY, UK; 3 Department of Horticulture, Faculty of Technical and Human Sciences, Sapientia Hungarian University of Transylvania Târgu Mureş/Corunca, Romania

**Keywords:** Population dynamics, Agroecology, Ecological networks, Biodiversity

## Abstract

Arthropods from four genetically modified (GM) maize hybrids (coleopteran resistant, coleopteran and lepidopteran resistant, lepidopteran resistant+herbicide tolerant and coleopteran resistant and herbicide tolerant) and non-GM varieties were sampled during a two-year field assessment. A total number of 363 555 arthropod individuals were collected. This represents the most comprehensive arthropod dataset from GM maize, and together with weed data, is reasonable to determine functional groups of arthropods and interactions between species. Trophic groups identified from both phytophagous and predatory arthropods were previously considered non-target organisms on which possible detrimental effects of *Bacillus thuringiensis* (*Bt*) toxins may have been directly (phytophagous species) or indirectly (predators) detected. The high number of individuals and species and their dynamics through the maize growing season can predict that interactions are highly correlational, and can thus be considered a useful tool to assess potential deleterious effects of *Bt* toxins on non-target organisms, serving to develop biosafety risk hypotheses for invertebrates exposed to GM maize plants.

## Background & Summary

This works presents the data underlying the studies in published papers^1–3^. In the USA, genetically modified (GM) maize was grown on 38 M ha in 2016 (ref. 4). Throughout EU countries maize is one of the major crops and is cultivated annually on approximatively 13 million hectares, representing 13% of the total cultivated area in the EU and about 8% of maize production area worldwide^5^. The two most potent insect pests of non-GM maize crops in Europe are the western corn rootworm (*Diabrotica virgifera virgifera* LeConte, Coleoptera: Chrysomelidae) and the European corn borer (*Ostrinia nubilalis* Hübner, Lepidoptera: Crambidae), the control of which represents the greatest challenge in European maize production^6^. Other insects, such as aphids (Aphidoidea) and thrips (Thysanoptera), also occur in all maize croplands and may occasionally contribute to crop losses^7–10^. Several arthropod predators preying on these and other arthropod herbivores are also found in maize croplands, the most important of which are ground beetles (Coleoptera: Carabidae), ladybird beetles (Coleoptera: Coccinellidae), rove beetles (Coleoptera: Staphylinidae), predatory thrips (Aleolothripidae), wolf spiders (Areneae: Lycosidae), Syrphid larvae (Diptera: Syrphidae) and minute pirate bugs (Orius spp.)^5^. While several previous studies have assessed the effects of GM maize toxins (especially *Bt* toxins) on non-target (particularly predatory) arthropods^3,11–16^, few studies have focussed on the strength of species’ trophic interactions and associated parameters among GM maize systems. This lack of knowledge represents a major issue in the analyses of arthropod communities associated with different GM plant species^17^.

Arthropod community studies in GM crop systems must involve a comprehensive and multi-methodological field assessment based on different collection methods of all arthropods and weeds from several different GM and non-GM control crops, over several years and throughout the maize growing season. Such datasets may allow us to assess the functional diversity of arthropod communities in GM and non-GM crops to be analysed at the same time, providing consistent species abundance and prey preference data^1,3,18^. However, datasets with hundreds of thousands of arthropod individuals from GM maize and its non-GM controls are rare, with no such datasets being freely available until now. The analysis of such data may demonstrate that interactions between species are highly correlated, and can thus be considered a useful tool in assessing the deleterious effects of *Bt*, and other toxins on a wide range of non-target organisms in GM croplands^1,3^. These data can also be a useful tool to develop biosafety risk hypotheses for invertebrates exposed to GM plants^18–20^. During a two-year intensive field assessment, detailed arthropod collections were made on different GM maize varieties (some are novel with no previous field test such as the coleopteran+lepidopteran resistant+glyphosate tolerant maize) and on their non-GM controls (Table 1).

## Methods

### Site characterisation and sampling procedure

**Figure 1:**
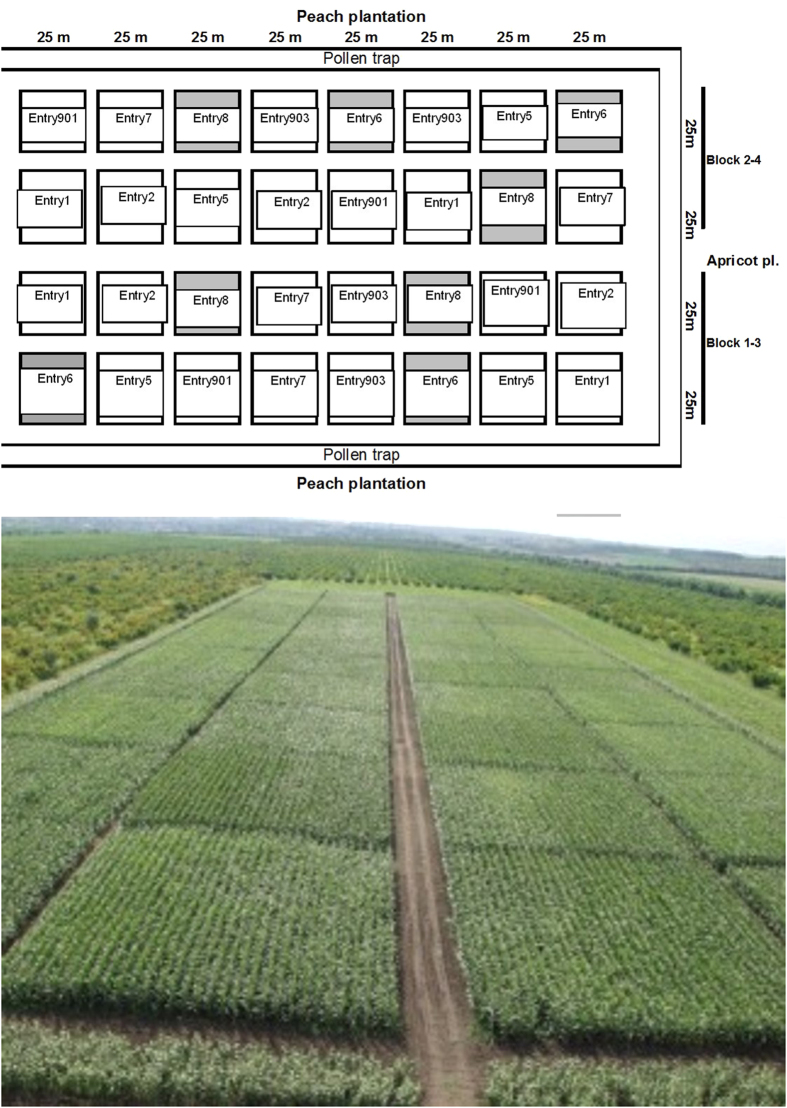
Experiment design of GM and control maize plots. Each block was planted in 625 m^2^ (25 m×25 m) plots spaced 3 m apart. Grey blocks represents extra glyphosate treatments applied. Treatments and replicates are presented in Table 1. Maize plants of similar maturity were established in the retention zone surrounding the experimental fields to capture pollen surrounding the entire experimental field site and prevent pollen contamination of non-GM fields in the wider area around the experimental fields. Part of the figure (field aerial photo only) has been published as supplementary online material in ref. 3 (ece32848-sup-0001-SupInfo.docx).

This section is an expanded versions of descriptions in our previous works^1–3^. The field sites were set up in 2007 in Central Europe, near Budapest, Hungary on chernozem soil in a completely randomised block design with each of the four different GM maize varieties and two non-GM controls. Peach and apricot orchards of about 200 ha dominated the area around the field sites, and no previous maize cultures were present in the immediate area, thus reducing the risk of cross-pollination by GM pollen as much as possible. Both GM and non-GM maize plots were established in 625 m^2^ (25 m×25 m) blocks, each spaced 3 m apart within each block (Table 1, Figure 1). The GM maize varieties tested contained proteins conferring resistance against two worldwide important maize insect pests (Western corn rootworm *D. virgifera virgifera* and European corn borer *O. nubilalis*), or an enzyme conferring glyphosate-resistance, or a combination of these. Tolerance of different GM maize varieties was conferred through different *Bt* insecticidal crystal (*Cry*) proteins in different forms that can specially target the above-mentioned two insect pests or the combinations of these. Two other GM maize varieties, in addition to insecticidal proteins, comprised of enzyme (C4 EPSPS) conferring tolerance to glyphosate herbicides (Table 1). Those GM maize varieties that did not contain glyphosate tolerances and the two non-GM controls were seeded in four replicates each. All glyphosate tolerant varieties were replicated eight times from which four blocks were subject to an extra glyphosate treatment in order to test and distinguish the effects of GM varieties vs. effects of glyphosate chemical application on arthropods (Table 1, Figure 1). The extra glyphosate in these blocks was applied in a total amount of 1060 g/ha each year at the four (V4) and eight leaf stages (V8) of the plants (according to the normal application procedures used in maize production). To prevent possible GM pollen contamination of non-GM fields in the wider area, non-GM maize plants of similar maturity were established in the retention zone surrounding the entire experimental fields as a measure to capture pollen. The same planting method, replication units, and glyphosate application were applied in the following year (2008). Maize was planted in late April 2007 and early May 2008, and harvested in late October 2007 and early November 2008. For two years (2007 and 2008), arthropods and weeds were sampled weekly from May, when plants reached the eight leaf stages (V8) until the end of the harvest period. Due to the relatively small block sizes, arthropods are likely to have moved between blocks, thereby reducing the impacts of the different treatments. As such, arthropod samples by pitfall and yellow trapping were taken only from a 10 m×10 m area in the centre of each 625 m^2^ block. In this way there was a distance of approximately 18–20 m between traps of different blocks. Three standard sampling methods were used to assess and collect arthropods:

1. Ground-dwelling arthropods were sampled using pitfall traps. Three traps were placed in the middle of each block (a total of 96 traps in the 32 blocks) and arthropods caught in traps were kept in absolute (99.8%) ethanol until identification. The whole procedure was repeated and traps emptied weekly from May until October. All individuals collected were counted and identified to the lowest taxonomic level possible.
2. Plant canopy dwelling arthropods were collected weekly using standard 30 cm×20 cm Pherocon™ yellow sticky traps. Three traps per block (a total of 96 in the 32 blocks) were placed in the centre of each block. Traps were changed once weekly from May to October each year. Arthropods were counted and identified to the lowest taxonomic level possible.
3. Arthropods were also assessed by visual observations. Fifteen plants per block were randomly selected (other than those containing yellow traps) from the middle of each block (480 plants per one assessment) and all arthropods found were counted and identified to the lowest taxonomic level possible.
4. Weed plant species and their coverage were assessed in all blocks by visual surveys on a random 3×1 m^2^ area from the middle of each plot as 0-100% area of coverage/species. Weed assessment was made at four different growth stages of maize (eight leaves stage (V8), twelve leaves stage (V12), vegetative stage, tasseling (VT) and reproductive stage, milk (R3)).

### Data adjustments

This section represents a simplified version of descriptions from our previous works^1,3^ and describes data adjustments before food web analyses. Arthropod food webs were built using several thousand individuals (lowest=24 972, highest=32 237) per GM maize type (sum of four replications/treatment). First, all arthropods collected were assigned to trophic groups, a widely accepted method in food web studies as it reduces methodological biases related to uneven resolution of taxa within and among species trophic relations^21^. Trophic groups were defined as taxa that share the same set of predators and/or prey. Before food web constructions, all possible predator-prey interactions of species identified in the GM and non-GM plots were carefully searched in previously published scientific literature. Trophic relations between species (or the next highest level of resolution available, usually genus) were checked using a total of 62 scientific references (Supplementary Table 1). The scientific nomenclature and taxonomy of every resource and consumer were standardised using the Global Names Resolver using the Global Biodiversity Information Facility dataset (http://resolver.globalnames.biodinfo.org/). Designation of trophic links followed methods presented by Gagic et al. 2011 and Jordán *et al.* 2012^21,22^. To increase the sensibility of the method, arthropod data was further adjusted according to the following parameters:

- Because maize pollen can move long distances by several ways (wind, mechanical, etc.), thereby posing a risk that non-GM maize fields might produce kernels with GM toxins (production of *Bt* toxins is a dominant trait), food webs of each GM and non-GM maize varieties were built using information on the trophic groups prior tasselling stage (VT) of maize.
- The presence and abundance of some, or all of the arthropods and weeds investigated varied significantly during the vegetation period. For example, the western corn rootworms adults were absent in April, May, June, present in July and August, and absent again in September, October and November. Therefore, food webs in each GM and non-GM maize varieties were constructed from arthropod and weed data when the abundance of the most frequent species was highest, but prior to pollen spreading (end of June, mid-July).

After adjustments, the food web parameters number of nodes (species), number of edges (trophic relations between species) and K indexes (keystone index) were calculated for each entry following Jordán *et al.* 2012^21^.

### Data Records

The data obtained is available as Data Citation 1. Sample 1_Pitfall Traps file contains arthropod data collected with pitfall traps; Sample 2_Yellow Traps contains data collected with yellow sticky traps; Sample 3_Plant Assessments contains data collected by plant surveys from genetically modified maize events expressing Cry34Ab1, Cry35Ab1, Cry1F and CP4 EPSPS proteins and controls. Values represent number of individuals, except for Sample 3_Plant assessment column, Spider Mites damages %, where the percentage of damages per single plant is given. All arthropod data (Sample 1, 2 and 3) were recorded during the maize developing stages (eight leaves stage (V8), twelve leaves stage (V12), vegetative stage, tasseling (VT) and reproductive stage, milk (R3)) and presented in second column in each dataset). Entry (column 3) describes treatment ID or different GM and non-GM maize varieties (i.e. Entry 1 means Coleopteran resistant GM maize; see Table 1). Replicates (Blocks) (column 4) describes replicates in each entry, along with the code in numbers (as in Table 1). Trap number (column 5) donates the number of traps reported in each entry and its replicates. In Sample 3_Plant Assessment dataset, instead of trap numbers the plant numbers in which arthropods were assessed are given. Sample 4_Weed coverage dataset contains weed coverage (%) in different maize growing stage periods. For weed data the first columns, ‘Sampling’ donates the sampling data made in each year. Columns 2, 3 and 4 contains the same data as described for the previous datasets (2 is Maize growing stage, 3 is Entries and 4 is Blocks). Column 5 (Sample (m2)) describes the number of visual sampling made on weed coverage in each block. Values for each weed species represents soil coverage from 0–100% in 1 m^2^ area. In food web graph indexes dataset, the number of nodes (species) and the number of edges (trophic relations between species) are presented for each entry. Values ‘in degree’ represents the number of trophic links with the species in the food web (number of other species feeding on this species). Values ‘out degree’ represents the number of trophic links between the species (number of other species eaten by the species). K indices are quantitative rank of species by their topological importance. As the K index increases, the species importance is greater in the functioning of the food web.

### Technical Validation

The results of food web analyses using the datasets reported were published in peer-reviewed journals^1–3^. The arthropod and weed data presented here and in online datasets have also been statistically analysed using standard statistical tools in the associated published papers. The datasets have been carefully checked for possible typing errors in the various published papers. If any discrepancies were found they were corrected before being placed on the web-resource. Weed data were identified to species-level. Arthropod data were identified to the lowest taxonomic level possible, as was the case for most of the predator species. All identified species (weeds and arthropods) were checked by the authors of the paper and by researchers from the Department of Plant Protection and Department of Entomology of the Szent István University, Hungary and by scientists from the Plant Protection Institute of the Hungarian Academy of Science. All datasets were cross validated by carefully checking all data from field sheets and any inconsistencies that might be introduced into the database by different team members were corrected. Additional consistency checks were carried out by carefully analysing the most appropriate scientific references and datasets published in order to establish species similarities found in the present assessment and previously published papers. In addition to these checks, each species identified to this level was checked according to its distribution map. This was done in order to ensure that there were no mistakes in species distribution (i.e. if certain species were indeed previously present in Central European agricultural crops). Necessary corrections and changes were made in the database.

### Usage Notes

The database contains information on the functional groups of arthropods from four different GM maize and its isogenic control. To our knowledge, this is the most comprehensive food web/weed/arthropod data assessment from GM maize to date. Future data analyses of the present data that needs further attention include:

- Weed data has not been analysed in detail in comparison with specific trophic groups or species in different GM crops and its control. As weed data has been collected in different maize growth stage periods (V8, V12, VT and R3), detailed arthropod abundance and/or analyses in different growth stages can be made and analysed.
- New methods of species trophic relations and associated metrics analyses can be made.
- Comparison with other arthropod abundances from other GM maize crops can be computed.
- Importance of specific non-target groups and their relative abundance on different GM maize has not been analysed.
- Data can be analysed using several other software applications’ (e.g., R, Matlab, SPSS).

## Additional information

**How to cite this work:** Pálinkás, Z. *et al.* Arthropods dataset from different genetically modified maize events and associated controls. *Sci. Data* 5:180019 doi: 10.1038/sdata.2018.19 (2018).

**Publisher’s note:** Springer Nature remains neutral with regard to jurisdictional claims in published maps and institutional affiliations.

## Supplementary Material



Supplementary Information

## Figures and Tables

**Table 1:** The characteristics of the investigated GM and non-GM (control) maize. This table presents the data underlying the studies in refs 1–3.

**Treatm. ID (Entry)**	**Replicates (blocks) and codes (numbers)**	**OECD Identifier**	**Toxin**	**Resistance/Tolerance**	**GM Yes or No**	**no. of blocks**
1	7, 13, 38, 43	DAS-59122-7	Cry34Ab1, Cry35Ab1	Coleoptera	YES	4
2	1, 23, 26, 31	DAS-01507-1xDAS-59122-7	Cry34Ab1, Cry35Ab1xCry1F	Coleoptera and Lepidoptera	YES	4
5	12, 17, 27, 37	DAS-01507-1xMON-00603-6	Cry1FxC4 EPSPS	Lepidoptera and glyphosate	YES	4
6	8, 21, 28, 45	DAS-01507-1xMON-00603-6	Cry1FxC4 EPSPS	Lepidoptera and glyphosate+glyphosate treatment	YES	4
7	3, 18, 29, 40	DAS-59122-7xDAS-01507-1xMON-00603-6	Cry34Ab1, Cry35Ab1xCry1FxC4 EPSPS	Coleoptera, Lepidoptera and glyphosate	YES	4
8	6, 19, 34, 36	DAS-59122-7xDAS-01507-1xMON-00603-6	Cry34Ab1, Cry35Ab1xCry1FxC4 EPSPS	Coleoptera, Lepidoptera and glyphosate+glyphosate treatment	YES	4
901	5, 15, 33, 44	PR-34A15	-	-	NO	4
903	2, 24, 25, 32	PR-35A30	-	-	NO	4
